# Exogenous Cry1Ab/c Protein Recruits Different Endogenous Proteins for Its Function in Plant Growth and Development

**DOI:** 10.3389/fbioe.2020.00685

**Published:** 2020-06-30

**Authors:** Jianmei Fu, Biao Liu

**Affiliations:** ^1^Nanjing Institute of Environmental Sciences, Ministry of Ecology and Environment, Nanjing, China; ^2^Institute of Plant Protection, Jiangsu Academy of Agricultural Sciences, Nanjing, China; ^3^College of Life Sciences, Nanjing Agricultural University, Nanjing, China

**Keywords:** Cry1Ab/c protein, protein interaction, subcellular co-localization, yeast two-hybrid assay, bimolecular fluorescence complementation, co-immunoprecipitation

## Abstract

Current risk assessments of transgenic crops do not take into consideration whether exogenous proteins interact with endogenous proteins and thereby induce unintended effects in the crops. Therefore, the unintended effects through protein interactions in insect-resistant transgenic rice merit investigation. Here, a yeast two-hybrid assay was used to evaluate interactions between *Bacillus thuringiensis* (Bt) protein-derived Cry1Ab/c insect resistance rice Huahui-1 and the endogenous proteins of its parental rice Minghui-63. The authenticity of the strongest interactions of Cry1Ab/c and 14 endogenous proteins involved in photosynthesis and stress resistance, which may be primarily responsible for the significant phenotypic differences between transgenic Huahui-1 and parental Minghui-63, were then analyzed and validated by subcellular co-localization, bimolecular fluorescence complementation and co-immunoprecipitation. As the exogenous full-length Cry1Ab/c protein was found to have self-activating activity, we cleaved it - into three segments based on its three domains, and these were screened for interaction with host proteins using the yeast two-hybrid assay. Sixty endogenous proteins related to the regulation of photosynthesis, stress tolerance, and substance metabolism were found to interact with the Cry1Ab/c protein. The results of bimolecular fluorescence complementation and co-immunoprecipitation verified the interactions between the full-length Cry1Ab/c protein and 12 endogenous proteins involved in photosynthesis 23KD, G, PSBP, Rubisco, Trx, THF1 and stress resistance CAMTAs, DAHP, E3s, HKMTs, KIN13A, FREE1. We used a combination of yeast two-hybrid, bimolecular fluorescence complementation, and co-immunoprecipitation to identify Cry1Ab/c interacting with rice proteins that seem to be associated with the observed unintended effects on photosynthesis and stress resistance between Huahui-1 and Minghui-63 rice plants, and analyze the possible interaction mechanisms by comparing differences in cell localization and interaction sites between these interactions. The results herein provide a molecular analytical system to qualify and quantify the interactions between exogenous proteins and the endogenous proteins of the recipient crop. It could help elucidate both the positive and negative effects of creating transgenic plants and predict their potential risks as well as net crop quality and yield.

## Introduction

Transgenic crops, also known as genetically modified crops, refer to crops and their descendants produced by DNA recombination technology to integrate exogenous genes (DNA sequences are introduced into target cells through genetic engineering or viral infection, etc.) into the genomes of recipient crops, in a way that is changing their genetic composition (Herdt, 2005). Transgenic crops have considerable potential economic, environmental, and social benefits. However, uncertainty regarding their unintended effects has been a major constraint in their commercial development and exploitation. Unintended effects in transgenic crops are difficult to evaluate in terms of their possible biosafety risks, and current research has failed to elucidate the molecular mechanisms responsible for these unintended effects.

It has been reported that exogenous genes in transgenic crops might induce unintended effects by disrupting host genes or rearranging DNA sequences at the insertion site (Forsbach et al., 2003), which causes somatic variation during the transgenic crop cultivation process (Labra et al., 2001; Noro et al., 2007), consuming excess host material and energy for their own expression (Zeller et al., 2010) as exogenous proteins interact with endogenous proteins in the recipient crops. However, there is currently insufficient empirical evidence to corroborate these theories. Although the crop cannot express naturally the exogenous proteins, the transgenic crops could express these proteins through integrating exogenous genes into the crop genome in all tissues throughout the growth stages (Fu et al., 2018), so these exogenous proteins might potentially interact with endogenous proteins to produce unintended biological effects in the transgenic crop. To date, however, the aforementioned issue has not raised sufficient concern. Moreover, the safety regulations and criteria for transgenic crops do not include an evaluation of the interactions between exogenous and endogenous proteins. Therefore, an analysis of the association between protein interactions and the unintended effects observed in insect-resistant transgenic rice is warranted.

The *Bt* gene from *Bacillus thuringiensis*, which encodes an insecticidal crystalline protein, is the most widely used insect-resistant gene in the world at present. Transgenic crops expressing Bt proteins can effectively control target insects, reducing the amount of required pesticides. On October 22, 2009, the Ministry of Agriculture of China issued biosafety certificates for the cultivation of the insect-resistant transgenic rice lines Huahui-1 and *Bt* Shanyou-63 in the Hubei Province, which were renewed in December 2014. However, the transgenic rice lines have not been cultivated commercially due to widespread concerns and controversy about its biosafety.

A large number of research results showed that there are many unexpected differences between Bt transgenic rice and its parent rice (Shu et al., 2002; Chen et al., 2006; Kim et al., 2008; Xia et al., 2010; Fu et al., 2018; Fu and Liu, 2020). Our previous study has reported that plant height, biomass, chlorophyll content, and other vegetative growth indicators are considerably different between insect-resistant transgenic rice Huahui-1 and the parent rice Minghui-63 under the farmland and saline-alkaline growth conditions (Fu et al., 2018). However, the molecular mechanisms underlying these phenotypic differences have yet to be determined. We speculate that exogenous Cry1Ab/c expressed in Huahui-1 interacts with endogenous proteins, alters rice metabolism and biological functions, and significantly changes the Huahui-1 phenotype. This will be based on exploring the molecular mechanism underlying the response to stress conditions via protein interactions. When crops are subjected to flooding, high salt concentrations, phosphorus deficiency, abscisic acid and other stressors, protein interaction-mediated oxidative stress responses occur that induce stomatal closure, decreases transpiration rates, and promote reactive oxygen species accumulation, among other processes (Wang et al., 2014; Singh and Sinha, 2016; Tang et al., 2016). Taoka et al. (2011) found that the Hd3a interacts with 14-3-3 proteins in the apical cells of shoots, yielding a complex that enters to the nucleus and binds to the OsFD1 transcription factor, resulting in the flowering by activating the flowering identity genes.

In a long-term study, we found that the differences in photosynthetic efficiency and stress resistance between the insect-resistant transgenic rice Huahui-1 and the parental rice Minghui-63 were most significant at the three-leaf stage (unpublished), but the molecular mechanism underlying these differences is still unknown. Referring to previous research-based speculation that protein interactions lead to rice flowering (Taoka et al., 2011), we hypothesized that Cry1Ab/c protein might interact with endogenous proteins in transgenic rice, which might have unintended effects on some biological characteristics, such as photosynthetic efficiency and stress resistance.

Therefore, the present study investigated exogenous Cry1Ab/c protein expressed by Huahui-1 with the following goals: (1) to screen potential endogenous proteins interacting with it. This would help to study the universality and intensity of the interaction between them; (2) to analyze and validate the interactions among Cry1Ab/c and endogenous proteins that are associated with the observed unintended effects on photosynthesis and stress resistance through subcellular co-localization, BiFC, and co-immunoprecipitation methods. This would help to establish a relationship between protein interactions and known phenotypic differences in photosynthesis and stress resistance; (3) to analyze the possible interaction mechanisms by comparing differences in cell localization and interaction sites between Cry1Ab/c and photosynthesis or stress resistance proteins. This information could contribute to identifying some unintended effects that might be caused by the expression of exogenous proteins, elucidating the possible molecular mechanisms through which protein interactions are associated with observed phenotypic differences between transgenic and parental crops. This would further provide guidance to improve the safety regulations and criteria for assessment of transgenic crops and to ensure the sustainability and healthy development of the transgenic crop industry.

## Materials and Methods

### Plant Materials and Growth Conditions

The rice varieties used in the present study were insect-resistant transgenic Huahui-1 containing the foreign *cry1Ab/c* fusion gene and parental Minghui-63, both of which were obtained from the College of Plant Science and Technology of Huazhong Agricultural University, Hubei, Wuhan, China. Large full seeds were surface-sterilized with 75% (v/v) alcohol, rinsed with ultrapure water, and placed in an unlit incubator at 30°C until germination. The seedlings were then transferred to a small pots and maintained in an incubator with a 16 h:8 h light:dark cycle at 28°C and 80% relative humidity (RH). At the three-leaf stage, the Huahui-1 and Minghui-63 rice lines showed substantial differences in plant height (Huahui-1, 16.38 ± 1.26 cm; Minghui-63, 19.82 ± 1.03 cm; *p* < 0.01) and fresh biomass (Huahui-1, 0.08 ± 0.00 g; Minghui-63, 0.12 ± 0.02 g; *p* < 0.01). Thirty Huahui-1 rice seedlings with equal growth rate were combined as a single sample and pulverized in liquid nitrogen in preparation for construction of a rice cDNA library.

*Nicotiana benthamiana* was cultivated in a greenhouse under a 16 h:8 h light-dark cycle, with a daytime temperature of 22–25°C and a nighttime temperature of 18–22°C. After 4–6 weeks (five-leaf stage), the plants were used in an *Agrobacterium* GV3101-mediated transient transformation experiment.

### Gene Cloning and Vector Construction

An RNeasy plant mini kit (Qiagen, Germany) was used to extract and purify RNA from Huahui-1 and Minghui-63 rice leaves, in accordance with the manufacturer’s instructions. One microgram of the extracted RNA was used as a template, and cDNA was synthesized from all samples by reverse transcription using a PrimeScript RT Reagent Kit with gDNA Eraser (RR047A; Takara, Japan). Foreign *cry1Ab/c* was amplified using Huahui-1 rice cDNA as a template. Endogenous genes were amplified using Minghui-63 rice cDNA as a template. The primers used for amplification are listed in Supplementary Table S1. The gene open reading frame sequences were acquired from the NCBI database (National Center for Biotechnology Information, Bethesda, MD, United States).

The recombinant plasmids used in the present study are listed in Supplementary Table S1. An In-Fusion HD Cloning Kit (TAKARA) reaction system was established to homologously recombine endogenous genes into the subcellular localization vector pBINPLUS-GFP, BiFC vector PCV-cYFP-N (nYFP), and yeast bihybrid vector pGBKT7 (BD) by using a single *Bam*H1 digestion site. The foreign gene *cry1Ab/c* was cloned into the subcellular location vector pBINPLUS-mCherry, BiFC vector PCV-nYFP-C (cYFP), and yeast two hybrid vector pGADT7 (AD).

### Screening for Endogenous Rice Protein Interactions With the Foreign Cry1Ab/c Protein by Yeast Two-Hybrid Assay

The yeast two-hybrid screening assays were conducted according to the instructions for the Matchmaker Gold yeast two-hybrid system (Clontech Laboratories, Mountain View, CA, United States). For large-scale extraction and identification of endogenous rice proteins interacting with the foreign Cry1Ab/c protein, the aforementioned cDNA library was inserted in the prey vector pGADT7 (AD) and the *cry1Ab/c* gene obtained from Huahui-1 cDNA template amplification was inserted in the bait vector pGBKT7 (BD). As the full-length *cry1Ab/c* gene self-activates, it was cleaved into three segments according to its three different domains. The three fragments were recombined in the bait vector BD and self-activation and cytotoxicity were verified. The vectors harboring the cDNA library and the aforementioned truncated genes were transformed into the yeast cells Y2H Gold and Y187, respectively, which were examined using double- and quadruple-deficiency screening assays. Positive colonies on the quadruple-deficiency screening plates were selected as templates and PCR amplification and sequencing were conducted to detect the inserted fragments. The NCBI database was consulted for gene identification.

### Transient Expression System Developed via *Agrobacterium*-Mediated Transformation

A transient expression system was developed based on *Agrobacterium*-mediated transformation as previously described (Zhang et al., 2011). For fluorescence observations, the tobacco leaves were excised and mounted on slides in water. Green fluorescence (GFP: 488 nm), red fluorescence (mCherry: 561 nm), and yellow fluorescence (YFP: 514 nm) were observed under a Zeiss LSM750 confocal laser-scanning microscope (Carl Zeiss AG, Oberkochen, Germany).

### Subcellular Co-localization

The target genes inserted into the pBINPLUS-GFP and pBINPLUS-mCherry vectors were transformed into *Agrobacterium* strain GV3101, which was infiltrated into *Nicotiana benthamiana* leaves. After 48 h, the GFP and mCherry fluorescence signals were read under a Zeiss LSM750 confocal laser-scanning microscope.

### Bimolecular Fluorescence Complementation

The target genes fused to the nYFP and cYFP vectors were transformed into *Agrobacterium* strain GV3101, which was infiltrated into *Nicotiana benthamiana* leaves (Li et al., 2019). As a positive control, we used 4A-nYFP (NbeIF4A) plus P2-cYFP (RSV-encoded proteins) (Shi et al., 2016). After 48 h, YFP fluorescence signals (514 nm) were observed using a Zeiss LSM750 confocal laser-scanning microscope.

### Co-immunoprecipitation Assays

The target genes inserted into the pBINPLUS-GFP and pBINPLUS-mCherry vectors were transformed into *Agrobacterium* strain GV3101, which was subsequently infiltrated into *Nicotiana benthamiana* leaves. After 48 h, 1.0 g tobacco leaves were pulverized with liquid nitrogen in a mortar. To enhance expression, the P19 strain was included in a co-transformation. Thereafter, 2 mL of extraction buffer was added and the homogenate was milled for 2 min. To the resulting homogenate, we added 50 μL of 20% (v/v) Triton [final concentration 0.5% (v/v)] and the macerate was ground for 1 min. The slurry was then transferred to a 2-mL Eppendorf tube. All samples were vortexed, mixed, and placed in a 4°C refrigerator, mixed by repeated inversions in a rolling-over instrument (Qilinbeier, Suzhou, China), and incubated for 20 min.

One milliliter of buffer (20 mM DTT, 1× proteinase, 50 mM Tris, 150 mM NaCl, 0.5 mM EDTA, and 8% glycerin) was added to a 1.5-mL Eppendorf tube and 35 μL of GFP-Trap A beads (ChromoTek GmbH, Planegg-Martinsned, Germany) was aspirated with a pipette cut off 3 mm from the tip and placed in the Eppendorf tube. The tube was centrifuged three times at 2,000 × *g* and 4°C for 4 min. At least 50 μL of liquid was retained in the tube after each run. The samples were centrifuged at 15,200 × *g* and 4°C for 5 min, and 1.3–1.4 mL of the resulting supernatant was transferred to a 1.5-mL Eppendorf tube, which was then centrifuged at 15,200 × *g* for 15 min. One hundred microliters of the resulting supernatant were used as the input, and the remaining 1–1.3 mL supernatant was added to the previously washed beads. The Eppendorf tubes were sealed with parafilm, placed in a 4°C refrigerator, mixed by repeated inversions in a four-dimensional rolling-over instrument (Qilinbeier, Suzhou, China), and incubated for 2 h. After a 3-min centrifugation at 2,000 × *g* and 4°C, the supernatant was removed and the GFP-trap A beads were washed six times with IP buffer and centrifuged at 2,000 × *g* for 1 min, so that the supernatant could be aspirated with a pipette. The remaining 80 μL of supernatant was combined with 5× SDS buffer and the mixture was heated to 95°C for 10 min and centrifuged at 2,000 × *g* and 20–25°C for 2 min.

Proteins were isolated using 4–20% prefabricated SDS-PAGE gels (Bio-Rad Laboratories, Hercules, CA, United States) in 1× running buffer. The proteins were then transferred to a polyvinylidene fluoride (PVDF) membrane using a Tris-Gly membrane transfer system (40°C; 40 V; 14–18 h). The membrane was thereafter blocked by immersion in 3% (v/v) bovine serum albumin and shaking at 80 rpm and 30°C for 2 h. The blocked membrane was washed with Tris-buffered saline (TBS) for 3–5 min, and then subjected to immunoblotting in an iBind western blot device (Invitrogen, Carlsbad, CA, United States) with 1× iBind Flex solution (100× additive, iBind Flex 5× buffer, and pure water). The primary antibodies used were anti-GFP (1:2,000) and anti-mCherry (1:1,500) tag (Abcam, Cambridge, United Kingdom), whereas sheep anti-rat (1:2,000; Abcam, Cambridge, United Kingdom) and Marker HRP (1:1,000; Bio-Rad, CA, United States) were used as secondary antibodies. The membrane was incubated with enhanced chemiluminescence (ECL) color developer and enhancement solution (1:1 mixture) for 5 min, and the colored bands that developed on the membrane were viewed using a VersaDoc imaging system (Bio-Rad Laboratories, Hercules, CA, United States).

## Results

### Yeast Two-Hybrid Screening for Interactions Between Exogenous Cry1Ab/c and Endogenous Rice Proteins

#### Detection of Self-Activation and Cytotoxicity in the Bait Yeast Strain

**FIGURE 1:**
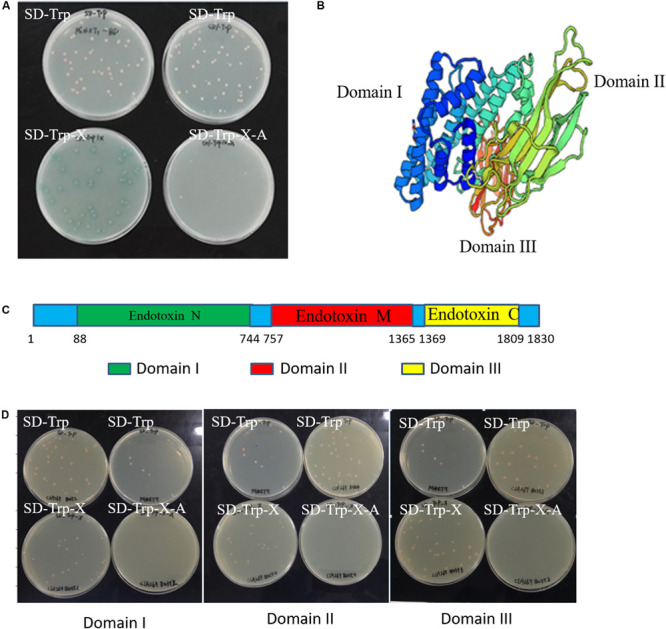
Self-activation and cytotoxicity detection in the Cry1Ab/c bait yeast strain. **(A)** The growth of full-length Cry1Ab/c-BD yeast colonies on nutrient-deficient SD/-Trp, SD/-Trp/X medium supplemented with x-alpha-gal (X) and SD/-Trp/X/A medium harboring strong abaR inhibitors; **(B)** A three-dimensional model of the full-length Cry1Ab/c foreign protein was predicted using Swiss-model online software (Adang and Crickmore, 2014); **(C)** A schematic diagram of the nucleic acid sequence sites for initiation and termination which were cut into three domains; **(D)** The growth of bait Domain I-BD, Domain II-BD and Domain III-BD yeast colonies on nutrient-deficient SD/-Trp, SD/-Trp/X, and SD/-Trp/X/A, respectively.

To screen the endogenous proteins interacting with Cry1Ab/c protein, it is necessary to detect self-activation and cytotoxicity of the Cry1Ab/c protein. Our results showed that the Cry1Ab/c-BD yeast strain Y2HGold bearing the full-length Cry1Ab/c protein developed normally on nutrient-deficient SD/-Trp medium, but turned blue on SD/-Trp/X medium supplemented with x-alpha-gal (X). The strain was also able to develop normally on SD/-Trp/X/A medium containing strong aureobasidin A (Figure 1A). Cry1Ab/c-BD presented with self-activation activity, as its double-reporter aur*1-C* and *mel1* could be induced. Therefore, the full-length Cry1Ab/c could not be used as bait for the subsequent prey protein screening.

A three-dimensional model of the full-length Cry1Ab/c exogenous protein was analyzed through Swiss-model online software. The results showed that it comprises three different domains, each of which might have unique functions (Figure 1B). Thus, referring to the instructions of the “Yeastmaker^TM^ Yeast Transformation System 2 User Manual,” the amino acid sequence of the target protein was truncated to prevent self-activation during yeast two-hybrid assay. The full-length Cry1Ab/c protein was accordingly cleaved into three segments based on the aforementioned Domains I–III in the present study (Figure 1C). The gene sequences of the three domains were cloned separately and fused with an expression vector PGBKT7 (BD) to generate three Domain-BD constructs, and the yeast strain Y2HGold was transformed to analyze self-activation. The three Domain-BD strains did not exhibit self-activation as they grew normally on SD/-Trp and SD/-Trp/X without turning blue and they failed to grow on SD/-Trp/X/A. There were no significant differences among the three Domain-BD yeast strains grown on solid or in liquid SD/-Trp in terms of growth status or rate. Thus, the expression of the three bait Domain-BD proteins in yeast was not toxic to host cells (Figure 1D) and these were used to screen Cry1Ab/c-interacting endogenous proteins.

#### Screening Cry1Ab/c-Interacting Endogenous Proteins

**FIGURE 2:**
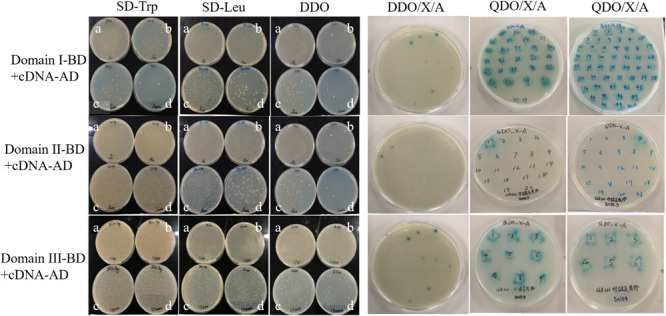
Cry1Ab/c-interacting endogenous proteins were preliminarily screened. The growth of three co-transformed Y2HGold yeast harboring Domain-BD and the Y187 yeast strain harboring cDNA-AD on nutrient-deficient SD/-Trp, SD-Leu, DDO, DDO/X/A, and QDO/X/A, respectively. a-d, the dilution ratios is 1/10, 1/100, 1/1000, 1/10000, respectively.

Three truncated domains of Cry1Ab/c mentioned previously did not result in self-activation and cytotoxicity, and thus, they were used as bait proteins to screen a rice cDNA library via a yeast two-hybrid approach. The three co-transformed Domain-BD + cDNA-AD yeast strains at four different dilutions grew normally on SD/-Trp, SD/-Leu, and SD/-Trp/-Leu (DDO). Thus, the Y2HGold yeast harboring Domain-BD and the Y187 yeast strain harboring cDNA-AD were successfully paired. Fifty plates were coated with DDO/X/A for blue-white and resistance screening. Seventy-five Domain I, 41 Domain II, and 24 Domain III blue positive clones were screened. The blue positive clones derived from the double nutrient-deficient DDO/X/A medium were transferred to a quadruple nutrient-deficient SD/-Ade/-His/-Leu/-Trp/X/A(QDO/X/A) medium. Seventy-four Domain I, 19 Domain II, and two Domain III blue positive clones were then screened. The earliest growth and deepest color changes observed indicated strongly interacting proteins, and these colonies were preserved as key material for further research (Figure 2). The blue positive clones were amplified by PCR, and those containing single fragments were isolated from the plasmid library and sequenced at the 5′ end after gel electrophoresis (Supplementary Figure S1). Having removed the repeat sequences, 60 candidate positive clones were designated as Cry1Ab/c-interacting endogenous proteins.

Based on their physiological and biochemical properties, the clones were classified as stress proteins involved in resistance, functional proteins involved in biomass and energy metabolism, photosynthetic proteins involved in oxidation-reduction reactions, and certain proteins of unknown function (Table 1). Our earlier research results revealed that the observed differences between the insect-resistant transgenic rice Huahui-1 and its parent rice Minghui-63 in terms of photosynthetic efficiency and stress resistance were the most pronounced at the three-leaf stage (unpublished), and thus, the Cry1Ab/c-interacting proteins associated with the aforementioned phenotypic differences were screened using the yeast two-hybrid assay at this stage. Therefore, in the present study, Photosynthesis and stress resistance proteins exhibiting the strongest interactions with the Cry1Ab/c protein were selected for analysis and to authenticate these protein–protein interactions through subcellular co-location, BiFC, and co-IP methods (Table 2).

**TABLE 1:** Detailed gene names and reference numbers of positive clones that are interacting with the Cry1Ab/c protein.

Functional classification	Name of protein interacting with Cry1Ab/c	Accession number
**Stress response**	Chromatin structure-remodeling complex protein SYD isoform X2	XM015786971.1
	Patellin-3	XM015784751.1
	Proline synthase co-transcribed bacterial homolog protein isoform X2	XM015777991.1
	Protein SGT1 homolog	XM015766221.1
	Proliferating cell nuclear antigen	XM015771759.1
	HSP70	L32165.1
	Bifunctional nuclease 1	XM015765834.1
	AP-1 complex subunit mu-2	XM015782448.1
	Putative wall-associated receptor kinase-like 16	XM015778712.1
	Peptidyl-prolyl cis-trans isomerase FKBP12	XM015769882.1
	ERBB-3 BINDING PROTEIN 1	XM015785210.1
	Protein TSS	XP 015646988.1
	Protein AE7-like 1	XM015772391.1
	Putative calcium-transporting atpase 8, plasma membrane-type (Ca^2+^-ATPase)	XP015649799.1
	V-type proton atpase subunit E-like (V-H^+^-ATPase)	XP015621799.1
	**Calmodulin-binding transcription activator 3 isoform X3 (CAMTAs)**	**XP015614398.1**
	**E3 ubiquitin-protein ligase RLIM (E3s)**	**XP015637952.1**
	**Kinesin-13A (KIN13A)**	**XP015639990.1**
	**Phospho-2-dehydro-3-deoxyheptonate aldolase 2 (DAHP)**	**XP015646378.1**
	**Chaperone protein dnaj-like isoform X1 (DnaJ)**	**XP015639177.1**
	**Histone-lysine *N*-methyltransferase family member (HKMTs)**	**XP015647562.1**
	**Protein FREE1 isoform X2 (FREE1)**	**XP015643101.1**
**Oxidation reduction (photosynthesis)**	**Oxygen-evolving enhancer protein 2 (PSBP)**	**XM015791242.1**
	**Chloroplast 23 KD polypeptide of photosystem II (23KD)**	**EU325990.1**
	**PITH domain-containing protein (Trx)**	**XM015775004.1**
	**Putative photosystem II component CP43 (CP43)**	**DQ336882.1**
	**Protein thylakoid formation1, chloroplastic (THF1)**	**XM015791425.1**
	**Ribulose-1,5-bisphosphate carboxylase/oxygenase large subunit, chloroplastic (RuBisCO)**	**NP039391.1**
	**Gtpase activating protein 1-like (G)**	**XM_015772115.1**
Material metabolism	Coatomer subunit delta-2	XM015784192.1
	Mitochondrial outer membrane protein porin 1	XM015756376.1
	Enolase-like	XM015777125.1
	Nicotiana attenuata eukaryotic initiation factor 4A-6	XM019379319.1
	Carbon catabolite derepressing protein kinase	EF575881.1
	Synaptotagmin-2	XM015756696.1
	Peptidyl-prolyl *cis*-*trans* isomerase FKBP12	XM015769882.1
	Seipin-1	XM015763551.1
	Exocyst complex component SEC5B	XM015780441.1
	Glutamine–tRNA ligase	XM015784471.1
	Suppressor of mec-8 and unc-52 protein homolog	XM015783294.1
	Probable protein *S*-acyltransferase 7	CP018170.1
	Knotted-1-like 13	XM015794843.1
Others	Predicted	XM015764852.1
	Predicted	BAS86484.1
	40S ribosomal protein S20-2-like	XM014805724.1
	Predicted	EU959177.1
	40S ribosomal protein S20	XM015787564.1
	Predicted	CP018163.1
	Predicted	–
	Probable DNA primase large subunit	XM006657577.2
	Predicted	XM015789372.1
	Predicted	XP015646911.1

*The bold black font indicates the candidate endogenous protein screened for further verification of the authenticity of interaction, and “–” indicates that the reference number has not been searched in NCBI.*

**TABLE 2:** Comparison between the bimolecular fluorescence complementation (BIFC) and co-immunoprecipitation (co-IP) assays in terms of the interactions between Cry1Ab/c and 14 endogenous proteins involved in photosynthesis and stress resistance.

		Differences between	
Protein classification	Protein name	BIFC and co-IP	
		BIFC	co-IP
Photosynthesis	23KD	Y	Y
	Trx	Y	Y
	THF1	Y	Y
	G	Y	Y
	PSBP	Y	Y
	RuBisCO	Y	Y
	CP43	Y	N
Stress tolerance	CAMTAs	Y	Y
	DAHP	Y	Y
	HKMTs	Y	Y
	KIN13A	Y	Y
	FREE1	Y	Y
	E3s	Y	Y
	DnaJ	N	N

*“Y” indicates Yes, “N” indicates No.*

We suggested that the results obtained might contribute to identifying the interactions that most likely account for the significant differences between transgenic rice Huahui-1 and parental rice Minghui63 in terms of photosynthetic efficiency, stress resistance, and other traits at the three-leaf stage.

### Validation of the Interactions Between the Exogenous Cry1Ab/c Protein and Endogenous Proteins

#### Subcellular Co-localization

**FIGURE 3:**
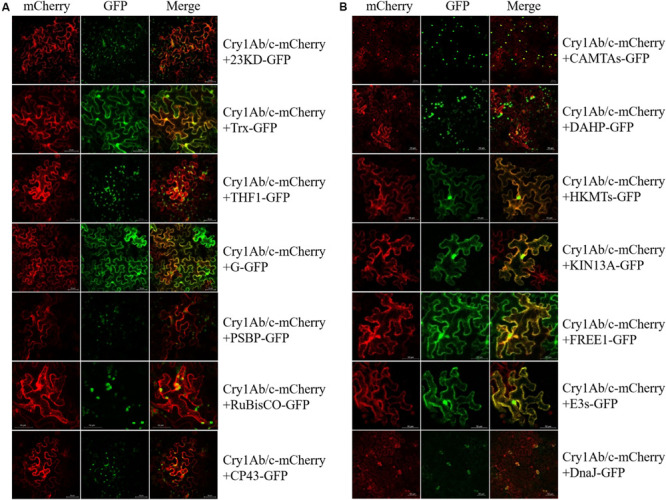
Subcellular co-localization of Cry1Ab/c and 14 endogenous proteins involved in photosynthesis and stress resistance. The co-localization sites and strength of photosynthetic proteins **(A)** and stress resistance proteins **(B)** were detected by *in situ* mCherry and GFP fluorescence, scale bars: 50 μm.

To determine the likelihood that these two different proteins would interact in the plant cell, the mCherry fluorescent protein-linked full-Cry1Ab/c protein and GFP-linked endogenous proteins were co-expressed in tobacco mesophyll cells. As shown in Figure 3, the exogenous full-length Cry1Ab/c-mCherry protein was mainly distributed in the cytoplasm and nucleus and should have therefore been able to interact extensively with the endogenous rice proteins. We found that the full-length Cry1Ab/c-mCherry protein and photosynthetic proteins 23KD-GFP, THF1-GFP, PBSP-GFP, and CP43-GFP co-localized in the chloroplast, with G-GFP mainly co-localized in the cell cytoplasm and Trx-GFP co-localized in the cell cytoplasm and nucleus. Additionally, the full-length Cry1Ab/c–mCherry protein and stress resistance proteins CAMTA-GFP, HKMT-GFP, KIN13A-GFP, FREE1-GFP, E3s-GFP, and Rubisco-GFP co-localized in the cell cytoplasm and nucleus, with DAHP-GFP co-localized in the cell organelles and DnaJ-GFP co-localized in the stomata.

#### Bimolecular Fluorescence Complementation

**FIGURE 4:**
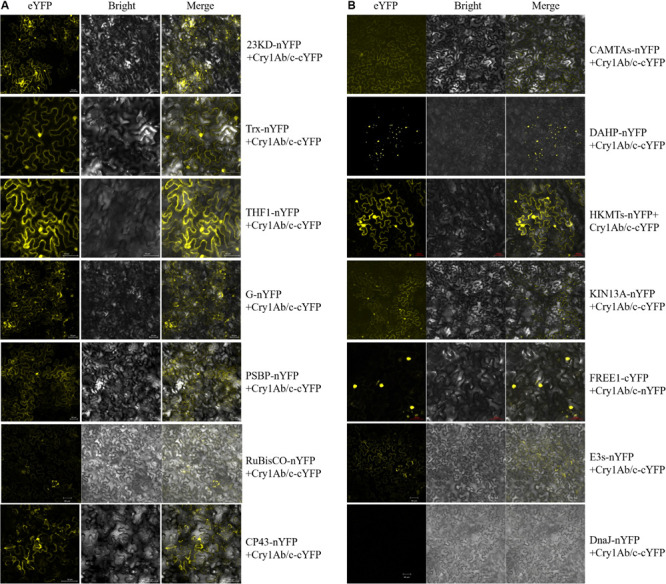
The bimolecular fluorescence complementation was used to verify interactions between the full-length Cry1Ab/c protein and 14 photosynthesis and stress resistance proteins. The sites and strength of interactions between the Cry1Ab/c protein and photosynthetic **(A)**, stress resistance proteins **(B)** were determined by *in situ* YFP fluorescence, scale bars: 50 μm.

To determine whether the exogenous full-length Cry1Ab/c protein and endogenous rice proteins do indeed interact in plant cells, the BiFC technique was performed. The expression vectors of full-length Cry1Ab/c protein and endogenous rice proteins fused to the N-terminal half of YFP and the C-terminal half of YFP, respectively, were then transiently expressed in tobacco mesophyll cells, and protein interaction sites and intensities were determined based on strong YFP yellow fluorescence. As shown in Figure 4, the protein interaction intensities differed between the full-length Cry1Ab/c protein and endogenous rice proteins. The full-length Cry1Ab/c-cYFP protein interacted strongly with 23KD-nYFP, PSBP-nYFP, G-nYFP, THF1-nYFP, and Trx- nYFP, but weakly with Rubisco-nYFP and CP43-nYFP. Similarly, the full-length Cry1Ab/c-cYFP protein interacted strongly with CAMTA-nYFP, DAHP-nYFP, HKMT-nYFP, KIN13A-nYFP and FREE1-nYFP, but weakly with E3s-nYFP. However, we detected no interaction between Cry1Ab/c and DnaJ-nYFP.

In addition, the protein interaction sites differed between the full-length Cry1Ab/c protein and endogenous rice proteins. An interaction between the full-length Cry1Ab/c-cYFP protein and photosynthetic proteins 23KD-nYFP, PSBP-nYFP, G-nYFP, THF1-nYFP, Trx-nYFP, Rubisco-nYFP and CP43-nYFP were observed in the cytoplasm and nucleus. An interaction between the full-length Cry1Ab/c-cYFP protein and stress resistance proteins CAMTAs-nYFP was observed in the cytoplasm, whereas DAHP-nYFP was observed in the organelle and HKMTs-nYFP, KIN13A-nYFP, and E3s-nYFP were observed in the cytoplasm and nucleus. FREE1-nYFP was observed in the nucleus.

#### Co-immunoprecipitation

**FIGURE 5:**
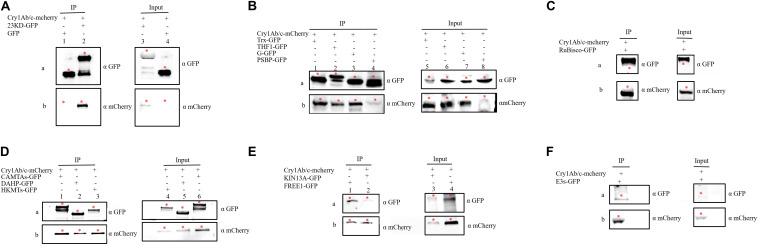
The co-immunoprecipitation was used to verify interactions between the full-length Cry1Ab/c protein and 12 endogenous proteins. Protein extracts (Input) were immunoprecipitated with GFP-trap beads (IP) and resolved by SDS-PAGE. The immunoblots shown were developed with anti-GFP antibody to detect the target endogenous protein involved in photosynthesis and stress resistance (a) and with anti-mCherry antibody to detect Cry1Ab/c (b, 94 kDa). **(A)** GFP + Cry1Ab/c-cherry (27 kDa, lanes 1 and 4), 23KD-GFP + Cry1Ab/c-cherry (47 kDa, lanes 2 and 3); **(B)** Trx-GFP + Cry1Ab/c-cherry (48 kDa, lanes 1 and 5), THF1-GFP + Cry1Ab/c-cherry(59 kDa, lanes 2 and 6), G-GFP + Cry1Ab/c-cherry (45 kDa, lane 3 and 7), PSBP-GFP + Cry1Ab/c-cherry (50 kDa, lanes 4 and 8); **(C)** Rubisco-GFP + Cry1Ab/c-cherry (80 kDa); **(D)** CAMTAs-GFP + Cry1Ab/c-mCherry (117 kDa, lanes 1 and 6), DAHP-GFP + Cry1Ab/c-mCherry (86 kDa, lanes 2 and 5), HKMTs-GFP + Cry1Ab/c-cherry (102 kDa, lanes 3 and 4); **(E)** FREE1-GFP + Cry1Ab/c-cherry (116 kDa, lanes 1 and 4), KIN13A-GFP + Cry1Ab/c-cherry (117 kDa, lanes 10 and 11); and **(F)** E3s-GFP + Cry1Ab/c-cherry (83 kDa, lanes 14 and 17). The red asterisk indicates the target band.

The interaction between proteins then needed to be confirmed using several methods, including co-IP. co-IP reflects protein interactions in the natural state of living plant cells, which can overcome the problem of false positives observed in BiFC that are attributable to spontaneous fluorescence (Speth et al., 2014). We found that the exogenous full-length Cry1Ab/c-mCherry protein interacted with the endogenous photosynthetic proteins 23KD-GFP, Trx-GFP, THF1-GFP, G-GFP, PSBP-GFP, and Rubisco-GFP (Figures 5A–C) but not with CP43-GFP (Supplementary Figure S2A); it can also interact with the endogenous stress-resistance proteins CAMTA-GFP, DAHP-GFP, HKMT-GFP, KIN13A-GFP, FREE1-GFP, and E3s-GFP (Figures 5D–F) but not with DnaJ-GFP (Supplementary Figure S2B). Differences between the BiFC and co-IP assays in terms of the interactions between Cry1Ab/c and 14 endogenous proteins involved in photosynthesis and stress resistance are listed in Table 2.

### Analysis of Possible Biological Functions Mediated by the Interactions Between Exogenous Cry1Ab/c Protein and Endogenous Proteins

To further analyze how the strong interactions between Cry1Ab/c protein and photosynthetic and stress resistant proteins affect biological phenotypes such as photosynthetic efficiency and stress resistance in insect resistant transgenic rice Huahui-1, we next compared the differences between subcellular localization or protein interaction sites of Cry1Ab/c and photosynthetic and stress resistant proteins and predicted their possible interaction mechanisms according to the results we identified. As shown in Table 3, the Cry1Ab/c protein was found to be mainly distributed in the cytoplasm and nucleus, with 23KD, THF1, and PSBP located in the chloroplast, whereas their interactions with the Cry1Ab/c protein were also mainly distributed in cytoplasm and nucleus. Thus, it was speculated that the expression of Cry1Ab/c protein might affect the function and photosynthetic efficiency of 23KD, THF1, and PSBP in the chloroplast by changing their cell location. Other photosynthetic proteins G and Trx are mainly distributed in the cytoplasm and nucleus, and their interactions with the Cry1Ab/c protein were also mainly distributed in the cytoplasm and nucleus. Therefore, it was considered that the interaction between large molecular Cry1Ab/c protein (67 Kda) and small molecular photosynthetic proteins G (18 Kda) and Trx (21 Kda) might inhibit the free movement of the latter in cells, thereby affecting photosynthetic functions of the latter.

**TABLE 3:** Differences between the location and interaction sites of Cry1Ab/c and 12 endogenous proteins involved in photosynthesis and stress resistance.

Protein	Protein	Differences between location	
classification	name	and interaction sites	
		Subcellular co-localization	BIFC
Photosynthesis	23KD	Chloroplast	Cytoplasm, nucleus
	Trx	Cytoplasm, nucleus	Cytoplasm, nucleus
	THF1	Chloroplast	Cytoplasm, nucleus
	G	Cytoplasm, nucleus	Cytoplasm, nucleus
	PSBP	Chloroplast	Cytoplasm, nucleus
	RuBisCO	Chloroplast	Cytoplasm, nucleus
Stress-tolerance	CAMTAs	Nucleus	Cytoplasm
	DAHP	Organelles	Organelles
	HKMTs	Cytoplasm, nucleus	Cytoplasm, nucleus
	KIN13A	Cytoplasm, nucleus	Cytoplasm, nucleus
	FREE1	Cytoplasm, nucleus	Nucleus
	E3s	Cytoplasm, nucleus	Cytoplasm, nucleus

*“BIFC” indicates “bimolecular fluorescence complementation.”*

For stress-resistance proteins, interactions with Cry1Ab/c were mainly located in the cytoplasm and nucleus, whereas CAMTAs are located in the nucleus and their interactions are mainly distributed in the cytoplasm. Thus, it was speculated that the latter might be transported out of the nucleus and detained in the cytoplasm for a long time, thus affecting the stress resistance and other biological functions of CAMTAs. Similarly, Cry1Ab/c and FREE1 proteins are mainly distributed in the cytoplasm and nucleus, whereas their interaction was distributed in nucleus, and thus, it was speculated that the interaction between Cry1Ab/c protein and FREE1 might inhibit shuttling of the latter from the cytoplasm to the nucleus, retaining it in the nucleus for a long time and thus affecting the stress resistance and other biological functions of FREE1. In contrast to the above the location and interaction sites of HKMTS and KIN13A, which were found to be distributed in the cytoplasm and nucleus, are consistent with the Cry1Ab/c protein. Thus, the underlying mechanism of how these interactions regulate plant stress resistance will need to be further studied through other molecular biological and physiological biochemical means in the future.

## Discussion

### Protein Interactions Might Comprise One of the Molecular Mechanisms That Cause Unintended Effects in Insect-Resistant Transgenic Rice

Previous studies have shown that comparisons of differences in agronomic traits (Xia et al., 2011; Wang et al., 2012a; Li et al., 2013; Jiang et al., 2016), nutritional composition (Wang et al., 2012b; Jian et al., 2014; Jiang et al., 2018; Takeda et al., 2019), environmental adaptability (Jiang et al., 2018; Wang et al., 2019; Park et al., 2019), gene transcription, protein expression, and metabolite levels (Liu et al., 2012; Liu Y. et al., 2015; Fu et al., 2019; Peng et al., 2019) among transgenic crops and their parents are important means and strategies for confirming and excluding the unintended effects of transgenic crops, in addition to facilitating any safety evaluations. However, the molecular mechanisms of underlying the unintended effects observed in transgenic crops are unclear.

Protein interaction networks play central roles in various life processes, such as signal transmission, gene expression regulation, energy and substance metabolism, and cell cycle regulation, and accordingly, it is assumed that the study of protein interactions will contribute to elucidating different biological functions in crops. Although the exogenous Cry1Ab/c protein is not produced naturally by the parent rice, it may be continuously expressed in all tissues during the entire crop growth period in insect-resistant transgenic rice Huahui-1, and thus, it might potentially interact with various endogenous rice proteins synthesized at different growth stages and have varying unintended biological effects. Taoka et al. (2011) found that Hd3a, 14-3-3, and transcription factor OsFD1 strongly interaction through yeast two-hybrid, GST pull-down and immunoprecipitation methods, and this interaction was found to result in rice flowering by inducing the expression of the downstream flowering gene. Similarly, Tang et al. (2016) found that OsbZIP46, a transcription factor that plays an important role in promoting ABA signal transduction and drought resistance in plants, and MODD exhibit strong interactions based on yeast two-hybrid, GST pull-down and co-IP methods; and this interaction inhibits the OsbZIP46 transcriptional activity and protein stability through recruiting other protein that regulate chromatin modifications and protein ubiquitin, respectively, thus suggesting an efficient and reasonable new mechanism of drought response. Therefore, an appropriate technology will enable the detection of these interactions between endogenous rice proteins and Cry1Ab/c protein, which could contribute to establishing the correlations between these protein interactions and the unintended biological effects in insect resistant transgenic rice.

The yeast two-hybrid screening approach used in the present study revealed 60 proteins that interact with Cry1Ab/c, including stress-resistance, photosynthetic, metabolic, and unknown proteins. Therefore, Cry1Ab/c and its interacting partners might comprise a platform for crosstalk among photosynthetic, stress resistance, oxidation-reduction, and other signal transduction pathways. Our findings that Cry1Ab/c might interact with various endogenous rice proteins are indicative of the explicit unintended effects at the protein levels after Cry1Ab/c proteins are expressed in transgenic rice.

### Methodological Discussion on Screening and Validating the Protein Interactions

In the yeast two-hybrid assay, certain bait proteins may induce the expression of reporter genes and self-activate, which limits their application to yeast two-hybrid technology (Tsuyoshi et al., 2002). An earlier study reported that the problem of bait protein self-activation can be resolved by introducing a deletion in the gene that removes the activation domain; hence, in this study, self-activation is re-assessed with respect to the remaining components, whereas the procedure of introducing a deletion in the gene could also inadvertently disrupt or remove a functional domain (Liu Y. L. et al., 2015). Wang et al. (2014) found the PHR2 N terminus has a transactivation domain that is active in yeast; therefore, in the Y2H assay, they used the PHR2 C terminus containing the MYB-CC domains (PHR2-C196aa) responsible for specific binding to the P1BS sequence.

In the present study, the initial yeast two-hybrid screening revealed that the full-length bait protein Cry1Ab/c could self-activate. Referring to the suggestion of “Matchmaker Gold Y2H system User Manual,” to retain the complete functional domains, Cry1Ab/c was cleaved into three segments based on its three functional domains, and we confirmed that none of them could self-activate. Therefore, the aforementioned system was able to be used to screen endogenous proteins interacting with Cry1Ab/c and could serve as a novel research tool for examining protein interactions in general. However, the original functionality would be lost or drastically impacted when the full-length Cry1Ab/c protein was divided into three segments to prevent self-activation during the yeast two-hybrid assay, thus screening the interacting proteins could also differ; therefore, the full-length Cry1Ab/c protein was used to verify the authenticity of the interactions with the endogenous proteins of interest through BiFC and co-IP methods.

Given that yeast and plant cells differ substantially in terms of protein modification, protein interactions within yeast cells might not accurately reflect those in plant cells. Therefore, two other technologies were applied to validate the protein interactions. Previous studies have authenticated protein interactions via comprehensive analyses based on yeast two-hybrid, BiFC, and co-IP assays (Taoka et al., 2011; Tang et al., 2016; Purwestri et al., 2017). These are also used to elucidate the underlying molecular mechanisms of the related physiological phenotypes. Ni et al. (2019) found that DMI3, as a positive regulator of abscisic acid signal transduction, it can interact with the rice protein phosphatase PP45 through yeast two-hybrid preliminary screening, and the authenticity of the interaction between the two proteins was verified in tobacco cells using co-IP and pull-down methods, which provided a research basis for elucidating the molecular mechanism of abscisic acid mediated stress resistance regulation in rice. Similarly, Purwestri et al. (2017) found that the flowering protein Hd3a can interact with certain rice proteins, including GF14c, OsKANADI and BIP116b through yeast two-hybrid preliminary screening, and the authenticity of the protein interactions was verified in tobacco cells by BiFC, furnishing clues regarding the molecular mechanisms underlying the regulation of rice flower signal transduction by Hd3a. Other similar studies have also preliminarily screened the plant endogenous proteins that can interact with a target protein through yeast two-hybrid approach, and the authenticity of protein interactions was verified in tobacco cells by BiFC and co-IP, which laid a research foundation that will contribute to clarifying the biological functions of target proteins in crops (Li et al., 2017; Shen et al., 2017; Ying et al., 2017; Wang et al., 2018).

Similarly, the Cry1Ab/c-interacting endogenous proteins were screened using a yeast two-hybrid, and the authenticity of the interactions between Cry1Ab/c and the screened endogenous proteins was verified in tobacco cells using BiFC and co-IP in the present study. These results provide a theoretical basis for clarifying the molecular mechanisms underlying the unintended effects that occur via protein interactions in transgenic rice.

### Validation of the Interactions Between Cry1Ab/c and Candidate Endogenous Proteins, and Analysis of Their Biological Functions

Our results showed that the differences between the insect-resistant transgenic rice Huahui-1 and the parental rice Minghui-63, especially photosynthetic efficiency and stress resistance, were most significant at the three-leaf stage in our long-term study (unpublished). Therefore, we want to further study the correlation between these phenotypic differences and protein interaction at the same three-leaf stage. All the methods used (yeast two-hybrid, BiFC and co-IP) clearly showed that the Cry1Ab/c protein and photosynthetic proteins 23KD, Trx, THF1, G and PSBP exhibit strong interactions. Since the Cry1Ab/c protein is mainly distributed in the cytoplasm and nucleus, whereas 23KD, THF1, and PSBP are localized in the chloroplasts, the mechanism of interaction of which the expression of exogenous Cry1Ab/c protein might inhibit the shuttling of 23KD, THF1 and PSBP from cytoplasm and nucleus into chloroplasts, which was confirmed by a BIFC experiment. This indicated that the interactions between Cry1Ab/c protein and 23KD, THF1, and PSBP might be explained by a model in which the expression of Cry1Ab/c protein could significantly affect the accumulation of the latter in the chloroplasts by changing their localization, thereby arresting photosynthesis. Kong et al. (2014) found that the interactions between the rice stripe virus SP protein and rice PsbP protein reduce the accumulation of the latter in the chloroplasts by changing its localization, thereby arresting photosynthesis and promoting foliar chlorosis, and these effects have been verified by PsbP mutation and overexpression assays. Other photosynthetic proteins G and Trx are mainly distributed in the cytoplasm and nucleus, and their interactions with the Cry1Ab/c protein are also mainly distributed in the cytoplasm and nucleus, which were confirmed by BiFC experiments. Thus, another possible mechanism that could explain the differences in photosynthetic efficiency caused by the expression of exogenous Cry1Ab/c protein is that the interaction between large molecular Cry1Ab/c protein (67 Kda) and small molecular photosynthetic proteins G (18 Kda) and Trx (21 Kda) might inhibit the free movement of the latter in cells, thereby affecting photosynthetic functions of the latter. Purwestri et al. (2017) found that GF14c protein, which causes delayed flowering, can interact with the flowering protein Hd3a, which inhibits the movement of the small protein Hd3a (about 20 Kda) from the leaf to the apical meristem (SAM), thus affecting the flowering of plants. Similarly, He et al. (2018) found that interactions between the rice fructokinase proteins OsFLN1 and OsFLN12 and thioredoxin OsTRXz (Trx) affected chloroplast development, which was verified by an OsTRXz mutation and overexpression assay. The above results indicated that the interaction between Cry1Ab/c proteins and photosynthetic proteins, such as 23KD and Trx, might be the main reason for the differences in photosynthetic efficiency between insect resistant transgenic rice Huahui-1 and its parent rice Minghui-63.

Among plant stress-related proteins, CAMTAs, HKMT, KIN13A, FREE1, and DAHP have been extensively investigated. These proteins regulate plant responses to high and low temperatures, drought, salinity, and other stressors, and in the present study, all the methods used (yeast two-hybrid, BiFC and co-IP) clearly showed that the Cry1Ab/c protein interacts strongly with these proteins in yeast and plant cells. Since the Cry1Ab/c protein is mainly distributed in the cytoplasm and nucleus, whereas CAMTAs reside in the nucleus, the mechanism underlying this interaction, wherein the expression of exogenous Cry1Ab/c protein might transport the latter out of the nucleus and retain it in the cytoplasm for a long time were confirmed by BIFC experiments. This would affect the stress resistance and other biological functions of CAMTAs. Ishida et al. (2004) found that 14-3-3 protein interacts with the leucine zipper structure transcription factor RSG in tobacco, which causes the latter to be retained in the cytoplasm to negatively regulate the GA (gibberellin) signaling pathway, and thus affecting the adaptability of plants to adversity. Similarly, Cry1Ab/c and FREE1 proteins are mainly distributed in the cytoplasm and nucleus, whereas their interaction is distributed in the nucleus. Therefore, another possible mechanism that could explain the differences in stress resistance caused by the expression of exogenous Cry1Ab/c protein is that the interaction between Cry1Ab/c protein and FREE1 might inhibit the shuttling of the latter from the cytoplasm into the nucleus, and retaining it in the nucleus for a long time, thus affecting the stress resistance and other biological functions of FREE1. A similar study found that plasma membrane-localized cold-response protein kinase 1 (CRPK 1) mediates 14-3-3 protein phosphorylation in the nucleus to regulate transcription factor CBF signaling, and thus negatively regulating plant responses to low temperature in *Arabidopsis thaliana* (Liu et al., 2017). Additionally, Cry1Ab/c and HKMTs and KIN13A proteins are mainly distributed in the cytoplasm and nucleus, and their interactions are distributed in the nucleus. Therefore, whether the interaction between Cry1Ab/c and HKMTs and KIN13A proteins might prevent the degradation of the latter by activating or inhibiting related enzyme activity or might promote or prevent modification of the latter. The mechanisms regulating plant resistance will need to be further studied through other molecular biological, physiological, and biochemical means in the future. The aforementioned results indicated that the interaction between Cry1Ab/c proteins and stress resistance proteins, such as CAMTAs and FREE1, might be the main reason for the differences in stress resistance between insect resistant transgenic rice Huahui-1 and its parent rice Minghui-63.

If the aforementioned photosynthetic and stress resistance proteins are affected, the photosynthesis and stress resistance responses themselves could also be modulated. Herein, expression of an extra exogenous Cry1Ab/c protein in transgenic rice Huahui-1 considerably affected the photosynthetic efficiency and stress resistance of this strain relative to those of parent rice MH63 (Fu et al., 2018), and accordingly, we propose that the observed unintended effects might be a result of the interactions between the Cry1Ab/c protein and endogenous photosynthesis and stress resistance proteins. However, not all interactions between Cry1Ab/c and interacting endogenous proteins induced phenotypic effects, and thus, future research should use other molecular biological, physiological, and biochemical means, such as transcriptome and proteome methods, to further explore the transcription and protein expression of aforementioned photosynthetic and stress resistance proteins that interact authentically and strongly with Cry1Ab/c. This might contribute to finding the real interacting proteins that affect the photosynthetic efficiency and stress resistance of transgenic riceHuahui-1, and thus, we posit that interactions between Cry1Ab/c and endogenous proteins comprise a possible cause of unintended effects in Huahui-1. This information will help to provide guidance to improve the safety regulations and criteria for transgenic crops and ensure the sustainability and healthy development of the transgenic industry.

## Conclusion

In this study, we focused on the exogenous Cry1Ab/c protein expressed in transgenic rice Huahui-1. An initial yeast two-hybrid screen revealed that the full-length bait protein Cry1Ab/c could self-activate. To retain the complete functional domain for an examination of its biological function, full-length Cry1Ab/c was cleaved into three segments based on three functional domains, to remove its activation domain. Thereby, the aforementioned system could be used to screen endogenous proteins interacting with Cry1Ab/c and might thereby serve as a novel research tool to investigate protein interactions. The yeast two-hybrid assay accordingly revealed 60 endogenous rice proteins that can interact with the Cry1Ab/c protein; BiFC and co-IP verified the interactions between the full-length Cry1Ab/c protein and 12 of the identified endogenous proteins involved in photosynthesis and stress resistance. Finally, the possible interaction mechanisms were analyzed by comparing the differences in cell localization and interaction sites between Cry1Ab/c and photosynthesis and stress resistance proteins. Collectively, our results highlight the importance of accurately interpreting the interactions between the exogenous Cry1Ab/c protein and endogenous rice proteins and identifying those interactions responsible for the observed unintended effects in insect-resistant transgenic rice Huahui-1. In the future, this information could be used to assess the effects of these protein–protein interactions on the crop yield and quality of insect-resistant transgenic rice, as well as those of other transgenic crops.

## Data Availability Statement

All datasets generated for this study are included in the article/Supplementary Material.

## Author Contributions

JF designed and carried out the experiments and wrote the manuscript. BL designed and helped to revise the manuscript.

## Conflict of Interest

The authors declare that the research was conducted in the absence of any commercial or financial relationships that could be construed as a potential conflict of interest.

## Supplementary Material

FIGURE S1 The selected positive insertions from the DH5α strains were detected by PCR.

FIGURE S2 Co-ip was used to verify interaction between Cry1Ab/c and photosynthetic protein CP43 and stress resistance protein DnaJ, respectively. Protein extracts (Input) were immunoprecipitated with GFP-trap beads (IP) and resolved by SDS-PAGE. The immunoblots shown were developed with anti-GFP antibody to detect target endogenous protein (a) and with anti-mCherry antibody to detect Cry1Ab/c (b, 94KD). **(A)** CP43-GFP+Cry1Ab/c-cherry (36KD); **(B)** DnaJ-GFP+Cry1Ab/c-cherry (76KD).

TABLE S1 Primers used for cloning the full-length gene based on subcellular co-localization and co-immunoprecipitation vectors.

## Abbreviations

23KD: chloroplast 23 kDa polypeptide of photosystem II
BiFC: bimolecular fluorescence complementation
CAMTAs: calmodulin-binding transcription activator
co-IP: co-immunoprecipitation
CP43: putative photosystem II component CP43
DAHP: 3-deoxy-D-arabinoheptulosonate-7-phosphate
DDO: double dropout medium SD/-Leu/-Trp
DDO/X/A: double dropout medium SD/–Leu/–Trp supplemented with X-a -Gal and aureobasidin A
DnaJ: chaperone protein dnaj-like isoform
E3s: E3 ubiquitin-protein ligase
ECL: enhanced chemiluminescence
G: Gtpase activating protein
HKMTS: histone-lysine *N*-methyltransferase family member
KIN13A: kinesin-like protein 13A
PSBP: oxygen-evolving enhancer protein
QDO/X/A: quadruple dropout medium SD/–Ade/–His–Leu/–Trp supplemented with X-a -Gal and aureobasidin A
Rubisco: ribulose-1,5-bisphosphate carboxylase/oxygenase large subunit
SD medium: synthetic dropout medium
THF1: thylakoid formation 1 protein
Trx: PITH domain-containing protein
X- α-gal: 5-bromo-4-chloro-3-indolyl- α-d -galactoside.
