# Supplementary material for: Exogenous Cry1Ab/c Protein Recruits Different Endogenous Proteins for Its Function in Plant Growth and Development
Source: Front Bioeng Biotechnol. 2020 Jun 30;8:685. doi: 10.3389/fbioe.2020.00685 (PMC7344169; doi:10.3389/fbioe.2020.00685)
Supplement: FIGURE S1 — The selected positive insertions from the DH5α strains were detected by PCR. [file Data_Sheet_1.pdf]

Supplementary information

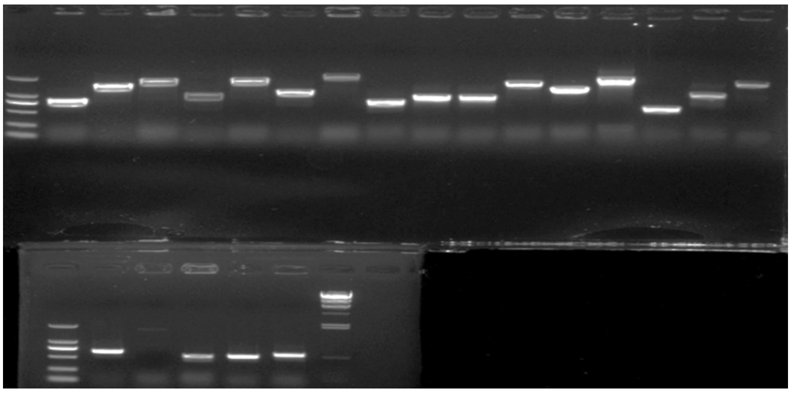

FIGURE S1 The selected positive insertions from the DH5α strains were detected by PCR.

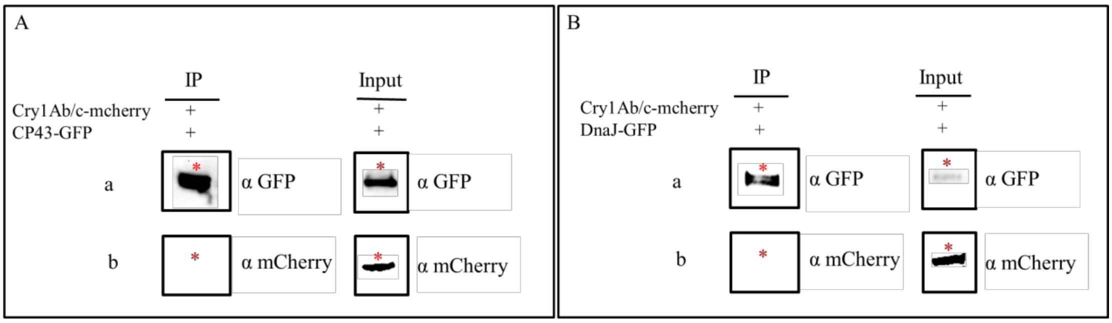

FIGURE S2 Co-ip was used to verify interaction between Cry1Ab/c and photosynthetic protein CP43 and stress resistance protein DnaJ, respectively. Protein extracts (Input) were immunoprecipitated with GFP-trap beads (IP) and resolved by SDS-PAGE. The immunoblots shown were developed with anti-GFP antibody to detect target endogenous protein (a) and with anti-mCherry antibody to detect Cry1Ab/c (b, 94KD). (A) CP43-GFP+Cry1Ab/c-cherry (36KD); (B) DnaJ-GFP+Cry1Ab/c-cherry (76KD).

Table S1 Primers for cloning the full-length gene based on subcellular co-localization and co-ip vectors

Primer name	Sequences
23KD-GFP4-F	CGGGGTCGACGGATCCATGGCGTCCACCTCCTGC
23KD-GFP4-R	TGCTCACCATGGATCCTGCGACGCTGAAGGAGC
Trx-GFP4-F	CGGGGTCGACGGATCCATGGCCGCCGCCACCA
Trx-GFP4-R	TGCTCACCATGGATCCATGCTCTTCTATCTTCTTCAGGTCC
THF1-GFP4-F	CGGGGTCGACGGATCCATGGCGGCCATATCTTCG
THF1-GFP4-R	TGCTCACCATGGATCCATGCCTCATGGAATTGAGACT
G-GFP4-F	CGGGGTCGACGGATCCATGTTGGGGCATCTGGTTG
G-GFP4-R	TGCTCACCATGGATCCTACACCCTTAGAACCTGGGAT
PSBP-GFP4-F	CGGGGTCGACGGATCCATGGCGTCCACCTCCTGCT
PSBP-GFP4-R	TGCTCACCATGGATCCTGCGACGCTGAAGGAGCTGGCTGCG
Rubisco-GFP4-F	CGGGGTCGACGGATCCATGTCACCACAAACAGAAACTAA
Rubisco-GFP4-R	TGCTCACCATGGATCCGCTATCTAGTTTATCTACCGGC
CP43-GFP4-F	CGGGGTCGACGGATCCATGACTCATGCTCCTTTAGGTTCTT
CP43-GFP4-R	TGCTCACCATGGATCCATTAAGAGGGGTCATGGAAA
CAMTAS-GFP4-F	CGGGGTCGACGGATCCATGCAGCAGCAGCAAAATGG
CAMTAS-GFP4-R	TGCTCACCATGGATCCGAAATAACCAGGGAGTGGTGTA
DAHP-GFP4-F	CGGGGTCGACGGATCCATGGCGCTCGCCACCAA
DAHP-GFP4-R	TGCTCACCATGGATCCGAAAGCCAATGGGGGCA
HKMTs-GFP4-F	CGGGGTCGACGGATCCATGGAGATGGACACATCGCC
HKMTs-GFP4-R	TGCTCACCATGGATCCCATGGTTAACTTTCCCACCCAT
KIN13A-GFP4-F	CGGGGTCGACGGATCCATGGGGGACTCCGGGGA
KIN13A-GFP4-R	TGCTCACCATGGATCCTCTGGAAGATTTCTTACGGCTG
FREE1-GFP4-F	CGGGGTCGACGGATCCATGCATCCCTCCGGTGAGTAC
FREE1-GFP4-R	TGCTCACCATGGATCCCAAAAACCAACTTGACTGGAAC
E3s-GFP4-F	CGGGGTCGACGGATCCATGGACAAATCAGAAGAGGATGC
E3s-GFP4-R	TGCTCACCATGGATCCTGTGGTTTTTCCAATGCCT
DnaJ-GFP4-F	CGGGGTCGACGGATCCATGGCGTTGCTACAGTTTGG
DnaJ-GFP4-R	TGCTCACCATGGATCCTCTCCTGCTGTTTGCTGTT
Cry1Ab/c-mCherry-F	CGGGGTCGACGGATCCATGGACAACTGCAGGCCATAC
Cry1Ab/c-mCherry-R	TGCTCACCATGGATCCTTCAGCCTCGAGTGTTGC

For bimolecular fluorescence complementarity vectors, the primer of homologous recombination of the underscore was replaced by CGGGAGATGCGGATCC (Forward primer) and GCTCGCCTGGGGATCC (Reverse primer) with termination codon.
